# A Pan‐European Whole‐Microbiome Study of Wastewater Influent: Prokaryotes, Protists, Fungi, and Metazoa

**DOI:** 10.1111/jeu.70112

**Published:** 2026-09-04

**Authors:** Niklas Nett, Kenneth Dumack

**Affiliations:** ^1^ Terrestrial Ecology, Institute of Zoology, University of Cologne Köln Germany; ^2^ Aquatic Ecosystem Analyses, Institute for Integrated Natural Sciences, University of Koblenz Koblenz Germany

**Keywords:** biogeography, community assembly, latitudinal gradient, microeukaryotes, wastewater treatment, WWTP

## Abstract

Microbial communities entering wastewater treatment plants (WWTPs) through untreated sewage represent an important interface between human, environmental, and treatment‐associated microbiomes, yet our understanding of their biogeography remains poorly resolved, particularly for microbial eukaryotes. Using shotgun metagenomic time‐series data from influent samples of seven WWTPs across a European latitudinal gradient, we analyzed the taxonomic composition and dynamics of bacteria, protists, fungi, and microscopic metazoa. Influent community composition varied with geographic location and season, with a pronounced north–south divergence driven by dominant taxa and stronger seasonal shifts observed at higher latitudes. Cross‐domain associations were pervasive, suggesting that co‐varying bacterial and eukaryotic components structure the incoming microbial pool. Our findings provide a pan‐European baseline for whole‐microbiome wastewater surveillance and highlight that influent communities differ regionally and seasonally. These patterns may be relevant for downstream treatment‐stage microbiomes, but direct effects on reactor community assembly and treatment performance require targeted sampling across treatment stages.

## 1 Introduction

Large volumes of water are used daily across households, industry, and agriculture, making wastewater treatment essential for environmental sustainability (Rodríguez et al. 2015; Pan et al. 2018). Before treatment begins, untreated sewage integrates microorganisms from human, animal, household, industrial, and environmental sources. This influent microbial community represents the biological input entering wastewater treatment plants (WWTPs) and provides a valuable matrix for studying microbial biogeography, temporal dynamics, influent communities in the community assembly of wastewater bioreactors, and wastewater‐based surveillance. Wastewater microbial communities are composed of well‐investigated prokaryotes and viruses, but also include functionally and epidemiologically relevant eukaryotes such as fungi, protists, and microscopic metazoa. In WWTPs, eukaryotic microorganisms do not only contribute to the degradation of organic matter but also play a key role in shaping the prokaryotic community structure through mechanisms such as bacterivory, grazing on biofilms, and competition for nutrients, ultimately enhancing treatment efficiency (Calaway 1963; Pauli et al. 2001; Peng et al. 2008; Freudenthal et al. 2022; Heck et al. 2023). Despite growing recognition of their functional importance, the environmental factors shaping microeukaryotic communities in WWTPs, and how the influent communities contribute to said community assembly, are still comparatively underexplored. To understand what these shaping factors might be, it is essential to consider how microbial communities are introduced and assembled within WWTPs.

In general, microbial communities in WWTPs originate primarily from microorganisms entering the plant through the influent, which, prior to reaching the facility, has already passed through shifts from aerobic to anaerobic phases. During this passage, the microorganisms are primarily subjected to strong deterministic selection, while stochastic effects likely play only a secondary role. As a result, only microorganisms capable of adapting to these fluctuating conditions survive, naturally assembling into a community within WWTPs shaped by the prevailing environmental filters (de Celis et al. 2022). It is well known that microbial communities in soils, lakes, and marine ecosystems vary along latitudinal gradients and in response to seasonality (Dolan 2005; Fierer and Jackson 2006; Ghiglione et al. 2012; Liu et al. 2015). Given that wastewater has already undergone strong filtering processes, it remains not well explored to what extent broader deterministic factors, such as geographic location and seasonal change, continue to shape the composition of microbial communities in WWTPs. While WWTPs have received comparatively little attention in this biogeographical context, emerging evidence suggests that microbial communities within them may exhibit geographic and seasonal structuring to some extent. Initial evidence comes from a global study of bacterial communities in activated sludge, the compartment in WWTPs where most microbial activity and degradation processes occur. This analysis revealed that only about 12% of operational taxonomic units (OTUs) form a shared core across WWTPs worldwide, while the majority of taxa exhibit substantial regional variation (Wu et al. 2019). One example of such regional variation is the occurrence of the filamentous bacterium *Trichococcus*, which was predominantly found in European WWTPs, while its abundance remained comparatively low on other continents. Similarly to Wu et al. (2019), a comparative study of WWTPs in Palestine, Israel, and Germany showed that microeukaryotic community composition in activated sludge also varies markedly between locations, with no evidence of a strong core community (Cohen et al. 2019). However, both studies relied on 16S or 18S rRNA‐based marker gene approaches, which are known to introduce strong methodological biases, particularly when assessing eukaryotic taxa. Those biases include variation in rRNA gene copy numbers between taxa (Not et al. 2009; Mäki et al. 2017), PCR amplification biases (Mathieu‐Daudé et al. 1996; Suzuki and Giovannoni 1996), primer mismatches due to the paraphyletic nature of protists (Martínez Rendón et al. 2025), as well as the use of different primer sets across studies, each favoring different taxa (Jeon et al. 2008; Elbrecht and Leese 2015). All of these biases may result in distorted abundance estimates and consequently affect the inferred community composition. In contrast, shotgun metagenomics offers a more robust alternative, eliminating primer and amplification biases and enabling a less distorted view on the microbial community composition, as shown by Freudenthal et al. (2022).

In this study, we made use of metagenomic data originally generated by Becsei et al. (2024), which includes influent samples from seven WWTPs distributed across Europe. While the original study focused exclusively on bacterial communities, we expanded this analysis to include eukaryotic microorganisms, such as protists, fungi, and microscopic metazoa. By investigating these previously understudied communities alongside bacteria using a cross‐regional approach, we aim to provide new insights into the structure, interactions, and shaping factors of microbial communities in wastewater. Particular emphasis is placed on identifying biogeographical patterns (latitude) and seasonal dynamics. Through systematically characterizing when and where specific taxa occur, this study provides a valuable reference point for future monitoring efforts and comparative research in wastewater microbiology. By systematically identifying when and where bacterial and eukaryotic taxa occur in untreated sewage, this study provides a baseline for future wastewater surveillance and for studies that explicitly track how influent communities are filtered across treatment stages.

## 2 Material and Methods

### 2.1 Data Access

**FIGURE 1:**
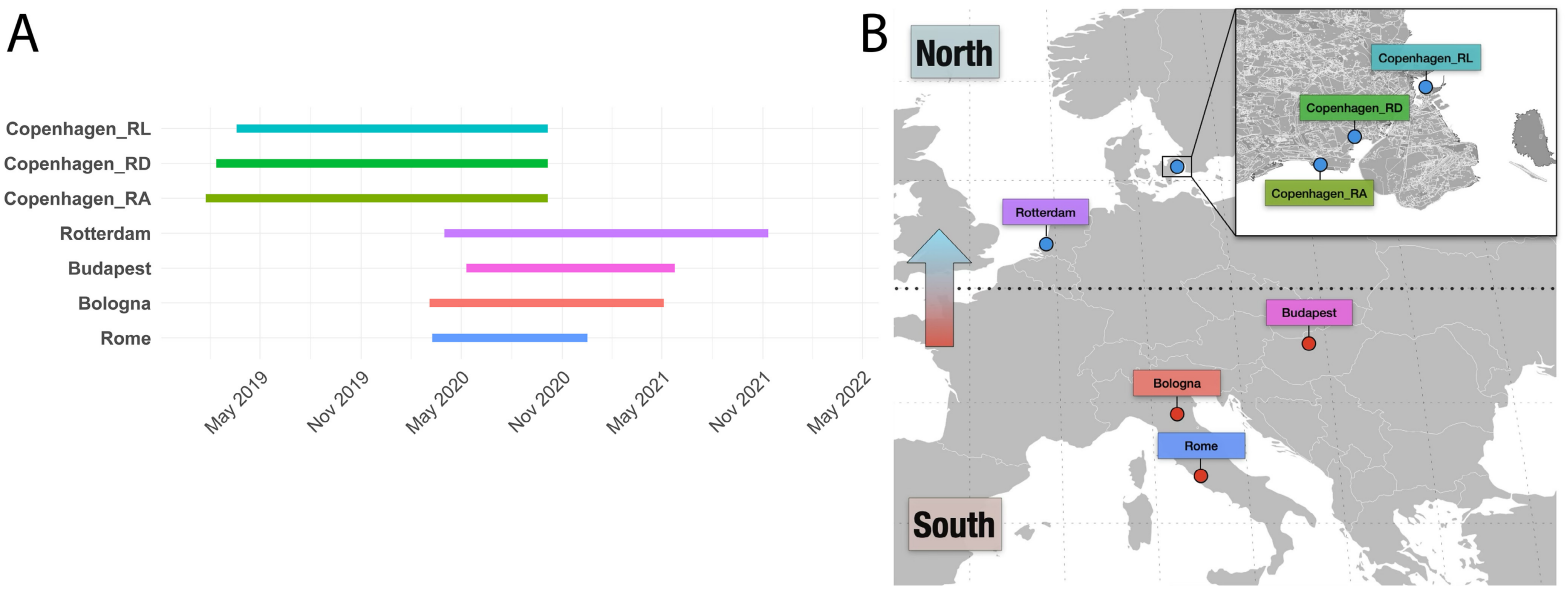
Sampling periods and geographic distribution of wastewater treatment plants (WWTPs) included in this study. (A) Colored bars indicate sampling durations for untreated sewage influent, based on data from Becsei et al. (2024). (B) Map of Europe showing all seven WWTPs arranged along a north–south latitudinal gradient. Northern plants are shown in blue, southern in red. A zoom‐in (top right) details the location of the three Copenhagen WWTPs, with connecting lines indicating their position on the main map.

For this project, data from the study by Becsei et al. (2024) was re‐analyzed. We accessed the sequenced data through the publicly available European Nucleotide Archive (ENA) under accession code PRJEB68319. Untreated sewage samples were collected across multiple WWTPs in Europe (Hungary, Denmark, Italy, and Netherlands). Sampling periods and locations are summarized in Figure 1. Detailed sampling dates for each plant, along with additional information, are provided in Table S1. In the Copenhagen plants, multiple replicates were occasionally collected on the same day. In this study, only one replicate per sampling date was included. Twenty‐four‐hour automatic continuous samplers were used to gather 1–2 L of sewage, except in Bologna, where three manual grab samples of 300 mL each were collected at 5‐min intervals between 8 and 10 AM and were subsequently pooled. The sewage was then frozen at −80°C and shipped to the Technical University of Denmark (DTU). Briefly, DNA was concentrated from frozen and thawed sewage by centrifugation, extracted, quantified and DNA libraries were prepared. The libraries were sequenced on the Illumina NovaSeq6000 platform, generating paired‐end reads with 150 base pairs per read direction (forward and reverse), resulting in a total read length of 300 base pairs per cluster. The sequencing targeted a minimum of 35 million clusters and produced approximately 10.5 Gbp of data per sample. For further details see Becsei et al. (2024).

### 2.2 Data Processing

Raw sequencing data, each with a read length of 150 bp, were quality‐checked using FastQC. Sample ERR12510732 was excluded from further analysis due to insufficient quality. All remaining samples showed a mean Phred quality score of ≥ 36, indicating consistently high read quality. Paired‐end reads (forward and reverse) were assembled into contiguous sequences using Mothur v1.45.3 (Schloss et al. 2009). Assembly parameters allowed a maximum of one error and a maximum ambiguity of 1 bp. However, a substantial proportion of reads failed to overlap, most likely due to library construction involving long fragments that were sequenced at only 150 bp. This design was compatible with the original intended pipeline, which employed MEGAHIT, a k‐mer‐based assembling tool optimized for fragmented short reads (Li et al. 2016). Due to limited computational resources and time constraints, an alternative approach was used for this study: to compensate for the high number of non‐overlapping reads, unassembled forward reads were extracted directly from the original FASTQ files and subsequently merged with the Mothur‐generated contig output data. This step allowed for the retention of a greater total number of sequences by accepting reads with a length of only 150 bp. The combined contig output, including the extracted forward reads, was subsequently screened for ribosomal RNA (rRNA) sequences using SortMeRNA v.4.3.4 (Kopylova et al. 2012). This step utilized several reference databases, including SILVA 16S, 23S, 18S, and 28S datasets. The resulting rRNA sequences were subjected to taxonomic assignment using BLASTN+ v.2.10.0 (Camacho et al. 2009). Eukaryotic sequences were screened against the PR2 v4.13.0 database (Guillou et al. 2013) and prokaryotic sequences were matched to the SILVA 138 Ref NR.99 database (Pruesse et al. 2007). Taxonomic assignments were refined by applying two filtering criteria: retaining only the best hit for each query sequence and setting an expectation value threshold of 1e‐30. The resulting BLAST output tables were further filtered to retain only microscopic metazoa (Nematoda, Rotifera, and Tardigrada), while excluding larger metazoan groups and plants (Embryophyceae), which were presumed to be remnants of decaying material within the wastewater rather than active community members. Additionally, sequences of the poorly annotated Rhogostomidae were merged under the genus name *Rhogostoma* for consistency, based on the results of Pohl et al. (2021). The microbial community was classified into prokaryotes (bacteria and archaea) and eukaryotes (protists, fungi, and microscopic metazoa). For convenience, in this paper, we refer to the assessed communities as “microbial communities” although they also include microscopic fauna such as nematodes, rotifers, and tardigrades. Following the nomenclature of the PR2 database, sequences that could not be assigned at the genus level were labeled according to the lowest assigned taxonomic rank (e.g., if only the order was identified, the genus was denoted as Ordername_X; if only the phylum was known, the genus was labled as Phylumname_XX). Read counts were calculated by summing all reads assigned to the same genus within each sample. Genera with five or fewer read hits were excluded to minimize background noise. All further diversity analyses were based on genus‐level assignments.

Data processing and manipulation were performed in R v.4.3.2 (R Core Team 2019), making use of the following packages: tidyr v.1.3.1 (Wickham et al. 2024), dplyr v.1.1.4 (Wickham et al. 2023), readr v.2.1.5 (Wickham et al. 2024), stringr v.1.5.1 (Wickham et al. 2023), tibble v.3.2.1 (Müller and Wickham 2023), lubridate v.1.9.4 (Grolemund and Wickham 2011), readxl v.1.4.3 (Wickham and Bryan 2023), writexl v.1.5.1 (Ooms 2017) and vegan v.2.6.8 (Oksanen et al. 2014). Visualizations were created using ggplot2 v.3.5.1 (Wickham 2016), ggbreak v.0.1.4 (Xu et al. 2021), ggnewscale v.0.5.0 (Campitelli 2024), and ggrepel v.0.9.6 (Slowikowski 2024). Geographic map data were retrieved from, including country boundaries (Admin 0—Countries) and graticule lines (Graticules 5°). A detailed inset map of Copenhagen was created using OpenStreetMap data, obtained via. WWTP locations were georeferenced and plotted using QGIS v.3.42.1 (QGIS Development Team 2025). Final figure arrangements were completed in Inkscape v.1.3.2. Rarefaction curves (Hurlbert 1971) were generated to assess whether sequencing depth was sufficient to capture the microbial diversity within each sample (Figure S1). The curves for the full dataset reached a clear saturation point, indicating that the sequencing depth was adequate to represent the microbial community. Separate rarefaction curves were generated for prokaryotes and eukaryotes (Figures S2 and S3). Both curves showed sufficient saturation, suggesting that neither community overshadowed the other in terms of sequencing coverage.

### 2.3 Influent Community Analyses

**FIGURE 2:**
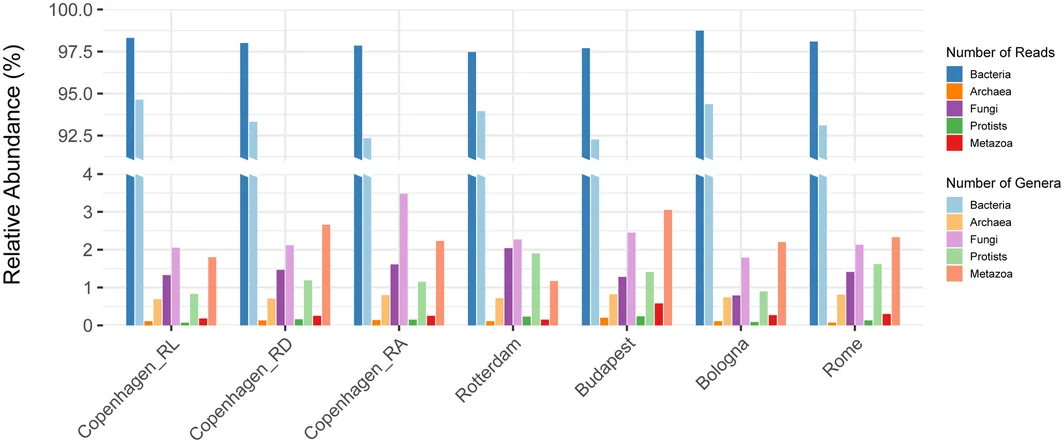
Relative read abundance and genus‐level diversity of major microbial communities across wastewater influents. Bar plots show the relative read abundance (upper panel, darker colors) and the proportion of assigned genera (lower panel, lighter colors) for five major microbial communities: Bacteria, Archaea, Fungi, Protists, and Metazoa. Data are presented for each WWTP influents for all time points, ordered geographically from north (Copenhagen_RL) to south (Rome).

To analyze the microbial community composition in the untreated sewage influent across WWTPs, a compound table was generated. For each plant, relative abundances for the number of reads assigned to each microbial community (Bacteria, Archaea, Fungi, Protists, and Metazoa) were calculated, along with the relative number of genera assigned per community. These values provided comparable estimates of community‐level diversity across sites (Table S2). The corresponding results are presented in Figure 2.

Archaea were excluded from all subsequent analyses due to their consistently low abundance and diversity across all plants, as well as their uniform distribution patterns observed in the compound table.

**FIGURE 3:**
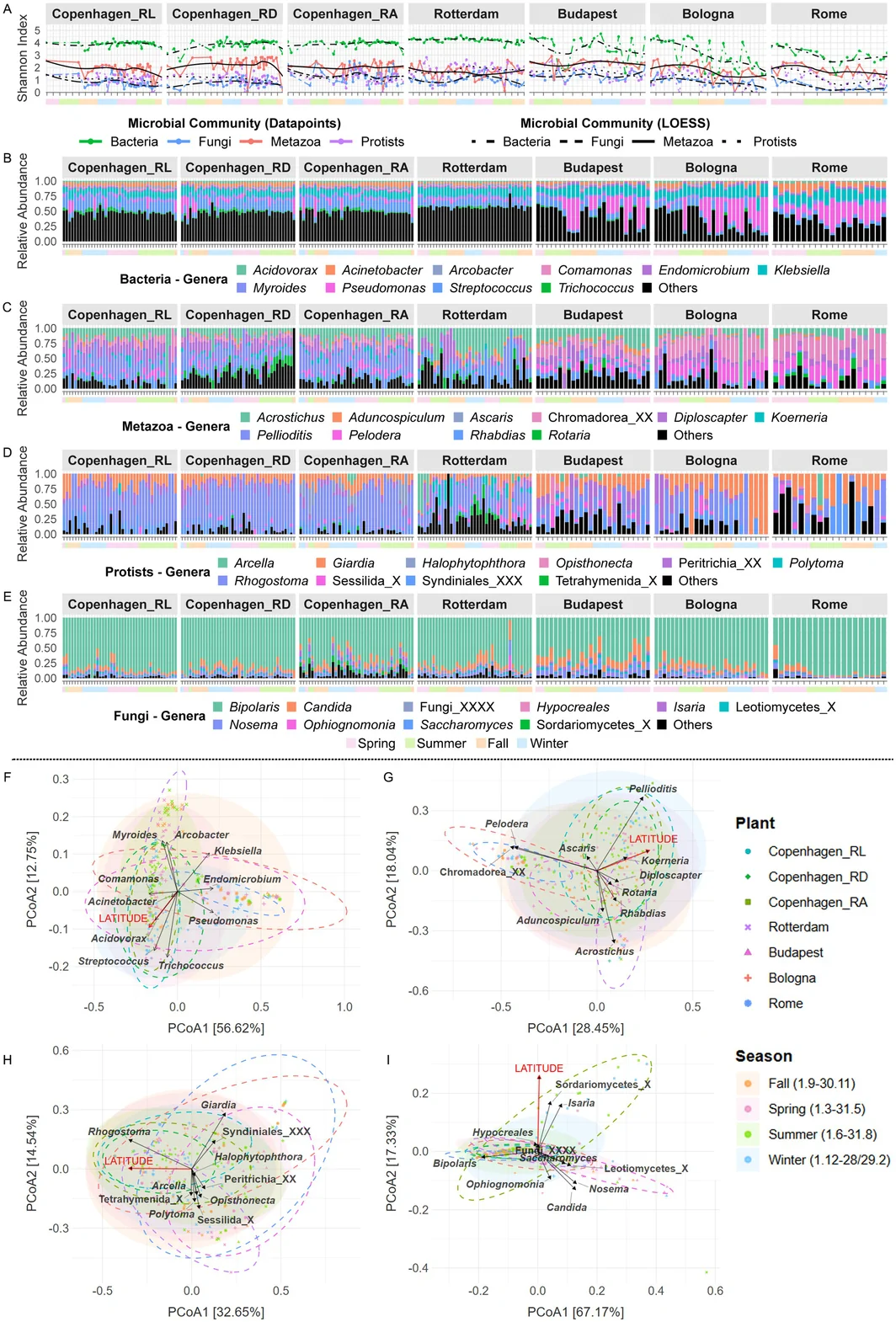
Overview of microbial community composition across European WWTP influents. The figure summarizes genus‐level diversity and composition within and across samples for microbial communities in seven European WWTP influents. (A) Alpha diversity is shown as Shannon‐Wiener indices for Bacteria, Metazoa, Protists and Fungi; each point represents a sample, and LOESS curves illustrate seasonal trends. (B–E) Relative abundance bar plots display the 10 most abundant genera per sample for each microbial community: (B) Bacteria, (C) Metazoa, (D) Protists and (E) Fungi. Remaining genera were grouped under “Others” (black), and each bar sums to 100%. Colored bars below each panel (A–E) represent meteorological seasons (legend shown beneath panel E). Samples are ordered geographically from north (Copenhagen_RL) to south (Rome). (F–I) Principal Coordinates Analyses visualize beta diversity patterns for (F) Bacteria, (G) Metazoa, (H) Protists, and (I) Fungi based on Bray–Curtis dissimilarities. Each point represents a sample, colored by meteorological season and shaped by WWTP of origin. Environmental vectors indicate sampling latitude (red arrows) and the top 10 most abundant genera per group (black arrows). 95% confidence ellipses were added for each plant and season.

For alpha diversity analyses, Shannon‐Wiener indices were calculated separately for each microbial community and sampling date across all WWTPs, capturing both richness (the number of distinct genera) and evenness (the distribution of reads among these genera) of the genus‐level community composition. The resulting indices were plotted against the sampling timeline for each plant, with smoothing curves added using LOESS (Locally Estimated Scatterplot Smoothing) regression to illustrate underlying temporal trends (Figure 3A). The corresponding values are provided in Table S3. To visualize relative abundances of microbial taxa within samples, stacked bar plots were generated (Figure 3B–E). Read counts were aggregated at genus level, and relative abundances were calculated by dividing each taxon's read count by the total number of reads assigned to the corresponding microbial community within the sample. This normalization allowed each bar to represent the full microbial community as 100% relative abundance, facilitating cross‐sample comparisons. The 10 most abundant genera per sample were displayed individually, while all remaining taxa were grouped under “Others.” The underlying values are listed in Table S3. To facilitate comparisons across WWTPs, mean relative abundances and standard deviations were calculated for selected genera based on this table. In addition, linear regressions were performed for taxa of particular interest to assess abundance changes along the latitudinal WWTP gradient from north to south, identifying potential biogeographic distribution patterns.

For beta diversity analysis, principal coordinates analysis (PCoA) was performed separately for each microbial community to identify overall compositional patterns and drivers of variation among samples (Figure 3F–I). Genus‐level read counts from all samples, including all seven WWTPs and sampling time points, were used to compute Bray–Curtis dissimilarity matrices, which then served as the basis for PCoA calculations. The data were projected onto two principal coordinate axes (PCoA1 and PCoA2), representing the main gradients of community composition. Samples were colored according to meteorological season, while the shapes of the datapoints indicate the WWTP of origin. 95% confidence ellipses were added for each plant and season to visualize potential grouping patterns. To identify potential drivers of community variation, environmental vectors were overlaid onto the ordination space. These included the sampling latitude (red arrow) and the 10 most abundant taxa from the same microbial community depicted in the corresponding PCoA plot (black arrows).

To statistically assess the drivers of beta diversity, PERMANOVA was conducted (Table S4). For each microbial community, genus‐level read counts were used to compute Bray–Curtis distance matrices. These matrices served as the response variables in PERMANOVA models evaluating the influence of the following explanatory factors: season, plant location, and latitude. To investigate potential cross‐community influences, the first principal coordinate (PCoA1) from each of the other microbial communities (originating from the prior performed PCoA calculations) were extracted and included as an additional explanatory factor in the respective PERMANOVA models. For example, in the model assessing bacterial beta diversity, PCoA1 scores of protists, fungi, and metazoa were used as predictors. PCoA1 represents the major axis of compositional variation and explained 56.62% of variation for bacteria, 28.45% for metazoa, 32.65% for protists, and 67.17% for fungi. For each term, PERMANOVA reports an *R* ^2^ value, representing the proportion of the remaining multivariate variance explained by the factor, and a *p* value, indicating its statistical significance (***p < 0.001; **p < 0.01; *p < 0.05; ·*p* < 0.10; ns *p* ≥ 0.10). The analysis was performed “by terms,” meaning that the *R* ^2^ value of each factor was calculated sequentially, so that after fitting the first factor, its explained variance was removed, and the next factor was evaluated based on the remaining variance. Since each plant is associated with a fixed geographic location, the factors plant location and latitude are inherently closely linked together. When plant location was modeled first, it absorbed most of the shared variance, causing latitude to become nonsignificant. To better capture the geographic gradient, latitude was therefore fitted before plant location in the PERMANOVA, despite this resulting in a less conventional order in which a factor with a lower *R* ^2^ (latitude) was evaluated before a factor with a higher *R* ^2^ (plant location). This approach was chosen to ensure that the effects of both factors could still be interpreted meaningfully, despite their strong co‐correlation. In addition, plant‐specific PERMANOVA analyses were performed to assess potential plant‐specific interactions between microbial communities and to further evaluate seasonal effects (Table S5). For each microbial community, genus‐level read counts were filtered by plant and used to compute Bray–Curtis distance matrices. These were then tested in PERMANOVA models using only season and the PCoA1 scores of the other microbial communities from the same site as explanatory variables. In general, the influence of seasonality is interpreted in the context of its complex ecological implications, as it represents a combination of environmental factors such as temperature and light availability. These factors can significantly affect microbial community structure and function in wastewater treatment systems, as suggested by previous studies (Kim et al. 2013; Liu et al. 2016; Heck et al. 2023).

All scripts and processed data that were used in this study, along with the Supporting Information tables, are available on GitHub at the following link: https://github.com/NiklasNett/whole‐wwtp‐microbiome‐europe. Artificial intelligence assisted in re‐writing this manuscript.

## 3 Results

### 3.1 Bacteria Dominate Read Abundance, While Eukaryotes Show Higher Genus‐Level Richness

The analysis of the metagenomic data from influent samples collected at seven European WWTP‐associated sites resulted in a total of 35,397,994 quality‐filtered sequence reads, which were assigned to 2141 distinct genera. Bacteria dominated both the read counts and genus‐level richness, accounting for 98.01% of all reads and 93.46% of all detected genera. Archaeal sequences represented 0.12% of all reads and 0.75% of all genera, and as stated in material and methods, are due to their low abundance not further considered in subsequent analyses. Among eukaryotes, fungi were the most abundant group, comprising 1.45% of all reads and 2.38% of all genera. Microscopic Metazoa represented 0.27% of total reads and 2.12% of genus‐level richness. Protists accounted for 0.15% of all sequence reads and 1.29% of all detected genera (Table S2). A closer examination of the community composition within each site revealed several plant‐specific deviations (Figure 2). Averaged across the entire sampling period, the Budapest plant exhibited the highest relative read abundances of both Archaea (0.58%) and Metazoa (0.20%), and also featured the greatest genus‐level diversity within the metazoan community at 3.05%. Along with Rotterdam, Budapest also exhibited the highest relative protistan read abundance, with values of 0.23% and 0.24%, respectively. However, Rotterdam surpassed all other sites in protistan genus‐level richness with a share of 1.90%, while Budapest, despite comparable read abundance levels, exhibited a genus‐level richness of only 1.41%. Additionally, Copenhagen_RA displayed the highest fungal genus‐level richness across the dataset at 3.48%, while all other sites showed values of 2.5% or less.

### 3.2 Geographic North–South Divergence in Bacterial Communities Is Driven by *Streptococcus* and *Pseudomonas*

To further investigate compositional differences across and within WWTP influents, microbial communities were analyzed in greater detail using Shannon diversity indices, relative abundance profiles, and Principal Coordinates Analysis (PCoA) (Figure 3).

Bacterial communities consistently exhibited the highest alpha diversity across all samples (Figure 3A). Northern WWTPs (Copenhagen_RA, Copenhagen_RD, Copenhagen_RL, and Rotterdam) maintained stable indices between ~4.0 and 4.5 throughout the year, with only minor temporal fluctuations, falling down to ~3.0 to 3.5 in some spring and fall samples. The overall stability in alpha diversity across northern WWTPs was reflected by a consistent distribution of dominant taxa throughout the year (Figure 3B), with the 10 most abundant taxa rarely exceeding 60% of the total community, indicating a high proportion of low‐abundance taxa. Northern WWTPs consistently showed contributions from *Streptococcus*, comprising approximately 10 to 25% of each sample. Short‐term drops in Shannon diversity, indicating a temporary reduction in evenness, were associated with blooms of *Pseudomonas*, which reached up to 32% relative abundance and coincided with a decrease in low‐abundance taxa. The compositional similarity among northern WWTPs was further reflected in the PCoA (Figure 3F). Samples from all three Copenhagen plants clustered tightly together. Although Rotterdam samples were spatially separated from the Copenhagen cluster along the PCoA2 axis, they remained aligned in a similar directional orientation. Northern samples were primarily associated with the environmental vector for latitude and the taxon *Streptococcus*.

In contrast, bacterial communities in southern WWTPs (Budapest, Bologna, and Rome) displayed pronounced seasonal variability in Shannon diversity, with indices fluctuating between ~1.5 and 4.5, including stronger declines and recoveries over time (Figure 3A). Lower diversities were often driven by short‐lived peaks in *Pseudomonas* dominance (Figure 3B), frequently exceeding 50% relative abundance within individual samples. The divergence in diversity profiles among southern WWTPs was also reflected in the PCoA (Figure 3F), where samples from Bologna, Budapest, and Rome clustered orthogonally to the northern WWTPs. Samples from Rome and Bologna overlapped extensively, while Budapest samples occupied a broader area between the northern and southern clusters. Rome and Bologna samples were closely associated with *Pseudomonas*. Geographical differences between northern and southern WWTPs were further supported by PERMANOVA, which identified latitude (*R* ^2^ = 20.75%, *p* = 0.001) and plant location (*R* ^2^ = 16.48%, *p* = 0.001) as significant drivers of bacterial community variation.

Seasonal effects were also apparent in the PCoA, where winter samples tended to cluster in the direction of the latitude vector. PERMANOVA confirmed seasonality as a significant driver of bacterial community composition, explaining 5.58% of the variation (*p* = 0.001).

### 3.3 Dominant Nematode Taxa Drive Regional Divergence in Metazoan Communities Between Northern and Southern WWTPs

Metazoan alpha diversity ranked second to bacterial alpha diversity across all WWTPs, with Shannon indices typically ranging from 1.5 to 3.0. These values exhibited marked temporal variation, characterized by abrupt increases and decreases throughout the year (Figure 3A).

All northern WWTPs shared similar profiles of dominant taxa (Figure 3C). In the Copenhagen plants, *Pellioditis* dominated most of the year, comprising approximately 20% to 45% of metazoan sequences in the majority of the samples, often accompanied by *Acrostichus* and *Diploscapter*. In Rotterdam, seasonal shifts were more pronounced, with *Acrostichus* and *Diploscapter* exhibiting distinct phases of dominance during summer and winter, reaching relative abundances of up to 54% and 58% of the metazoan sequences, respectively.

In contrast, the southern WWTPs (Budapest, Bologna, and Rome) exhibited different community profiles. Chromadorea_XX and *Pelodera* frequently emerged as dominant taxa, whereas *Pellioditis* was often absent. In Bologna and Rome, Chromadorea_XX maintained relative abundances of around or above 30% over extended time periods. *Pelodera* reached relative abundances of up to 63% in Bologna and 35% in Rome in certain samples. Budapest, on the other hand, showed less pronounced blooms of these taxa, with Chromadorea_XX generally remaining below 25% and *Pelodera* peaking at only 20%. This lower dominance of individual taxa in Budapest is also reflected by a more stable Shannon index over time. The compositional differences of metazoan communities between northern and southern WWTPs were also evident in the PCoA (Figure 3G). Samples from Copenhagen and Rotterdam shared a similar orientation, only being spatially separated along the PCoA2 axis. Copenhagen samples were primarily shaped by *Pellioditis*, while Rotterdam samples were influenced by *Acrostichus*, *Rotaria*, *Rhabdias*, and *Aduncospiculum*. Rome and Bologna formed a parallel but distinct cluster, largely structured by Chromadorea_XX and *Pelodera*. Budapest occupied an intermediate position but aligned directionally more with the Italian plants. These patterns were supported by PERMANOVA, which identified plant location (*R* ^2^ = 19.55%, *p* = 0.001) and latitude (*R* ^2^ = 17.85%, *p* = 0.001) as significant factors influencing the metazoan community composition.

Seasonal ellipses overlapped substantially in the PCoA, but exhibited subtle directional shifts. Winter samples clustered more closely with the Copenhagen communities, while spring and summer samples were distributed between northern and southern WWTPs. PERMANOVA identified seasonality as a significant driving factor of metazoan community variation, explaining 2.92% of the total variation (*p* = 0.001).

### 3.4 *Rhogostoma* and *Giardia* Drive North–South Structuring of Protist Communities

Shannon diversity plots of the protistan community revealed no clear spatial or temporal trends across all WWTPs (Figure 3A). However, the PCoA (Figure 3H) showed distinct biogeographic structuring, with clusters shaped by dominant taxa. Samples from Rome and Budapest clustered in a similar orientation, shaped primarily by *Giardia* and Syndiniales_XXX. Bologna appeared slightly offset but remained aligned with the southern WWTPs. Orthogonally to the southern WWTPs, samples from Rotterdam formed a separate cluster. Copenhagen samples clustered distinctly between Rotterdam and the southern WWTPs, aligning along the *Rhogostoma* vector, which itself closely followed the latitude vector. While *Giardia* occurred as a dominant taxon in samples across all WWTPs, its dominance was more frequently observed in southern WWTPs, aligning with the pattern found in the PCoA (Figure 3D). The regional disparity becomes particularly evident when comparing mean relative abundances, with *Giardia* reaching 21% ± 15% in Budapest, 38% ± 34% in Bologna, and 27% ± 20% in Rome, and only 9% ± 14% in Rotterdam, 14% ± 8% in Copenhagen_RA, 12% ± 7% in Copenhagen_RD, and 17% ± 12% in Copenhagen_RL. *Rhogostoma* also occurred as a dominant taxon throughout all WWTPs, but remained consistently dominant throughout the year in all three Copenhagen WWTPs, reaching a mean relative abundance of 53% ± 23% in Copenhagen_RA, 62% ± 18% in Copenhagen_RD, and 55% ± 21% in Copenhagen_RL. In contrast, *Rhogostoma* was frequently minor or entirely absent in Rome and Budapest, but maintained an average relative abundance of approximately 30% in both Bologna and Rotterdam.

PERMANOVA identified plant location (*R* ^2^ = 12.76%, *p* = 0.001) and latitude (*R* ^2^ = 10.83%, *p* = 0.001) as significant contributors to the protistan community composition. Regarding seasonal effects, the PCoA showed strong overlap among all seasonal ellipses. Nonetheless, the PERMANOVA still confirmed season as a variation contributor, but with a lower effect size (*R* ^2^ = 1.83%, *p* = 0.034).

### 3.5 *Bipolaris*‐Dominated Fungal Communities Display Low Diversity Across WWTPs

Among all microbial communities analyzed, fungal alpha diversity was consistently the lowest across all WWTPs and timepoints, with Shannon indices generally ≤ 1.5 and even < 0.5 in Rome (Figure 3A). This low diversity was primarily driven by the dominance of *Bipolaris* (Figure 3E), which prevailed in nearly all samples, particularly in Copenhagen_RL, Copenhagen_RD, Bologna, and Rome, where its average relative abundance ranged between 78% and 89% of the fungal sequences. Greater variability was observed in Budapest, Rotterdam, and Copenhagen_RA, where *Candida*, *Nosema*, and *Isaria* occasionally displaced *Bipolaris*, reaching relative abundances of up to 33%, 24%, and 13% in some samples, respectively.

This compositional homogeneity was also reflected in the PCoA (Figure 3I), where samples from Bologna, Rotterdam, Rome, and Copenhagen_RD/RL formed a central cluster, shaped predominantly by *Bipolaris* and *Saccharomyces*. In contrast, Copenhagen_RA formed a separate, vertically elongated cloud, influenced by Sordariomycetes_X, *Isaria*, and latitude. Winter samples tended to align more closely with the Copenhagen_RA cluster, whereas samples from other seasons clustered around the central group.

PERMANOVA results confirmed these patterns, identifying plant location (*R* ^2^ = 26.73%, *p* = 0.001), season (*R* ^2^ = 3.83%, *p* = 0.003), and latitude (*R* ^2^ = 2.04%, *p* = 0.005) as significant drivers of fungal community variation.

### 3.6 Plant‐Specific Seasonal Effects Reveal Stronger Community Shifts in Northern WWTPs

To assess the influence of seasonality at individual WWTP sites, separate PERMANOVA models were run for each plant and microbial community (Table S5).

While season was a significant driver of community variation in all northern WWTPs for bacteria (*R* ^2^ > 20%, *p* = 0.001–0.002), no such effect was observed in the southern WWTPs (Budapest: *R* ^2^ = 7.82%, *p* = 0.729; Bologna: *R* ^2^ = 12.05%, *p* = 0.391; Rome: *R* ^2^ = 31.46%, *p* = 0.051). Similarly, season significantly influenced metazoan communities in all northern WWTPs (*R* ^2^ > 10%, *p* = 0.001–0.049), whereas among the southern locations, a significant effect was observed only in Budapest (*R* ^2^ = 26.91%, *p* = 0.002).

For protists, seasonal variation was observed only in Copenhagen_RA (*R* ^2^ = 25.73%, *p* = 0.002) and Rotterdam (*R* ^2^ = 17.18%, *p* = 0.001). Fungal communities exhibited significant seasonal shifts exclusively in Copenhagen_RA (*R* ^2^ = 25.43%, *p* = 0.012) and Rome (*R* ^2^ = 53.03%, *p* = 0.001).

No significant seasonal influence was detected for any microbial community in Bologna.

### 3.7 Cross‐Community Associations Explain Additional Variation in Influent Microbial Composition

To further explore potential interactions between microbial communities and whether the composition of one community could explain variation in others, PERMANOVA analyses were extended and revealed several significant cross‐community associations (Table S4).

Bacterial composition significantly influenced the community structure of fungi (*R* ^2^ = 2.37%, *p* = 0.001), protists (*R* ^2^ = 1.44%, *p* = 0.003), and metazoa (*R* ^2^ = 0.69%, *p* = 0.018). Protists affected all other microbial communities, with the strongest influence on metazoa (*R* ^2^ = 1.13%, *p* = 0.001), followed by bacteria (*R* ^2^ = 0.96%, *p* = 0.018), as well as a trend to influence fungi (*R* ^2^ = 0.76%, *p* = 0.081). Fungal composition also significantly shaped the other communities, most notably bacteria (*R* ^2^ = 2.99%, *p* = 0.001), followed by metazoa (*R* ^2^ = 1.09%, *p* = 0.004), and protists (*R* ^2^ = 0.86%, *p* = 0.027). Metazoan communities only had a significant effect on protist composition (*R* ^2^ = 0.95%, *p* = 0.011). Additional plant‐specific interactions between microbial communities are summarized in Table S5.

## 4 Discussion

Characterizing influent communities may support wastewater‐based surveillance and provide a baseline for taxa entering treatment plants. The present study aimed to expand upon previous metagenomic research by incorporating not only bacterial but also eukaryotic communities in the analysis of the influent of seven WWTPs from different regions across Europe. This broader scope enabled a comprehensive characterization of microbial community composition across regionally distinct WWTP influents, allowing us to assess the consistency of taxonomic profiles between sites and to examine how geographic location (latitude) and seasonal factors shape community structure. By integrating both dimensions, the study demonstrates that microbial composition and dynamics in WWTPs vary with geography and season, suggesting that regionally stratified influent monitoring may be useful, and treatment‐stage studies are needed to test downstream relevance.

### 4.1 Latitude, Season, and Cross‐Community Interactions Jointly Shape WWTP Influent Microbial Community Composition

We observed that both geographic location (latitude) and season influence the structure of microbial communities in WWTP influents. These factors may reflect differences in source inputs and in‐sewer conditions before treatment, alongside stochastic influences. Our results revealed a pronounced biogeographical divide in microbial community composition between influent communities from northern and southern sites, observed across bacteria, protists, and metazoa. These results further support the observations made by Wu et al. (2019) and Cohen et al. (2019), which are now confirmed using a less biased metagenomic approach on influent data, indicating that microbial community composition varies geographically.

Seasonal changes, such as fluctuations in temperature, light availability, and humidity, can promote or suppress the growth of specific taxa, thereby reshaping community composition over time (Woombs and Laybourn‐Parry 1986; Wei et al. 2018). In addition, seasonal pathogen outbreaks may further contribute to compositional changes. For instance, a higher frequency of parasite infections during summer months, partly associated with increased recreational water use, can lead to temporary spikes of specific taxa Hlavsa et al. (2015).

Our Plant‐specific analyses revealed that seasonal effects on microbial community composition were most pronounced in northern WWTPs, whereas most southern WWTPs showed little or no significant seasonal variation. This difference may be attributed to the more extreme annual fluctuations in temperature and light availability observed in northern regions, which could impose stronger seasonal selection pressures on microbial communities. Therefore, our findings suggest that in regions with stronger seasonal shifts in microbial community composition, it may be particularly important to consider seasonal stratification in influent monitoring. Our results, which link higher latitude with increased seasonal effects on microbial communities, further suggest that the downstream relevance of regional influent differences should be tested across treatment stages.

In addition to latitude and season, our results indicate that interactions between microbial groups also serve as important shaping factors for all microbial communities. This highlights the value of our comprehensive approach, which considered multiple groups simultaneously. While bacteria accounted for the majority of microbial reads across all WWTPs, eukaryotic communities consistently exhibited higher genus‐level richness despite their lower read abundance (Figure 2). Previous research, such as Freudenthal et al. (2022), has shown that eukaryotic groups like protists can display high functional activity even when metagenomically low. Combined with our findings, this highlights the potentially strong role that eukaryotes may play in shaping microbial community structure. Protists, for example, likely influence bacterial and fungal composition through top‐down control, as many taxa are known bacterivores (Hahn and Höfle 2001) and also graze on fungi (Geisen et al. 2015; Estermann et al. 2023). In addition, our findings suggest that protists also shape the metazoan composition, possibly by mainly acting as intermediate trophic links, as both protists and nematodes feed on biofilms and may interact through shared prey resources (Freudenthal et al. 2022). In turn, bacterial and fungal communities appear to drive variation in both protist and metazoan composition, likely through bottom‐up effects driven by their abundance as prey. Additionally, our results indicate that bacterial and fungal composition are shaped by one another, possibly due to direct interactions such as competition for nutrients, metabolic cooperation in pollutant degradation (Mikesková et al. 2012), and mutual inhibition via antimicrobial compounds (Kerr 1999; Deshmukh et al. 2015). Metazoa showed limited effect on other communities, significantly affecting only the composition of protists, while being themselves shaped by all other groups. This asymmetry likely reflects their role as higher‐level grazers with slower dynamics and lower abundance. We further suggest that these interactions, acting as shaping factors, are themselves influenced by seasonal dynamics, indicating that biotic relationships are modulated by changing environmental conditions throughout the year. When examining the plant‐specific PERMANOVA results, we observed that cross‐community effects were more likely to be detectable in cases where seasonal variation also significantly influenced the tested community. This may suggest that seasonal changes not only alter individual community structures but also affect the strength and nature of their interactions. In particular seasons, the emergence of specialized taxa may intensify dynamic and fluctuating cross‐community interactions (Baltes et al. 2025). For example, Heck et al. (2023) showed, based on the investigation of a temperate WWTP in northern Europe, that predatory behavior of protists towards bacteria increases during seasonal conditions.

The effects of latitude and season in our study appeared to facilitate the dominance of specific taxa, as the observed biogeographical separation was primarily driven by shifts in the relative abundance of dominant groups, with certain taxa consistently associated with either higher or lower latitudes. This further underscores that distinct regions receive different influent taxa of potential operational or public‐health relevance. One aspect of such challenges is the region‐specific occurrence of pathogenic microorganisms. Our results show that within the bacterial community, *Streptococcus* was more dominant in northern WWTP influents, while *Pseudomonas* blooms were more prevalent in southern WWTP influents (Figure 3B). Both genera include human‐pathogenic strains that may carry clinically relevant antibiotic resistances (Odjadjare et al. 2012; Slekovec et al. 2012; Jiao et al. 2017), highlighting the importance of their effective removal. Within the protistan community, we also identified pathogenic taxa with distinct latitudinal distributions. Southern WWTP influents showed higher proportions of *Giardia*, whereas *Rhogostoma* dominated northern WWTP influents (Figure 3D). *Giardia* is a flagellated intestinal parasite transmitted via fecal contamination from infected humans or animals. Infected hosts can shed millions of cysts into the sewage, which are highly resistant to environmental stress and standard disinfection procedures (Ortega and Adam 1997). We detected particularly high *Giardia* abundances in Bologna and Rome, confirming earlier reports from Italian WWTPs by Cacciò et al. (2003). Besides parasitizing certain fish species (Dyková and Tyml 2016), *Rhogostoma* has recently been proposed as a potential host and vector of human‐pathogenic bacteria (Pohl et al. 2021). While it has already been recognized as a commonly found protist in wastewater systems (Pohl et al. 2021), our findings now also suggest that its abundance may be more likely found in WWTP influents of higher latitudes. Detecting region‐specific pathogenic taxa along a latitudinal gradient highlights the need to evaluate whether region‐specific influent taxa persist through treatment and affect disinfection requirements. Furthermore, pathogen surveillance in the influent may help track the progression and origins of outbreaks, thereby facilitating targeted prevention and improved containment strategies. For many WWTPs, sludge bulking represents a major operational problem. It is a process in which filamentous bacteria reduce sludge settleability and consequently impair treatment efficiency. In our study, the filamentous bacterium *Trichococcus*, commonly associated with sludge bulking (Wu et al. 2019), was more abundant in northern WWTP influents, with relative abundances ranging from 3% ± 2% in the north to nearly 0% in the south (Figure 3B). Because *Trichococcus* was detected in influent, monitoring may help identify incoming taxa associated with sludge‐bulking risk, but reactor colonization was not assessed. This gradient is supported by a significant negative correlation with latitude (*R* ^2^ = 25%, *p* < 0.001), indicating that *Trichococcus* abundance systematically decreases from north to south. This exemplifies that specific microbial challenges may be more prevalent in distinct geographic regions, with influent monitoring offering a potential early‐warning system to support preemptive treatment strategies. Our results further refine the global‐scale analysis by Wu et al. (2019), which identified increased *Trichococcus* abundances in European WWTPs, now specifying that its dominance is particularly pronounced in northern Europe. These findings highlight that even within the same continent, WWTPs may face region‐specific microbial challenges through the influent.

In addition to treatment challenging taxa, we observed that potentially beneficial taxa exhibit variation along a latitudinal gradient. Within the metazoan community, *Pellioditis* was dominant in Copenhagen influents, whereas *Pelodera* and unclassified Chromadorea were more abundant in southern influents such as Bologna and Rome (Figure 3B). All identified genera belong to the Chromadorea order and likely share similar functional traits. In WWTPs, these organisms may contribute to nutrient cycling and bacterial regulation by burrowing through biofilms, maintaining porosity, and thereby enhancing oxygen flow and reducing the risk of clogging (Calaway 1963; Woombs and Laybourn‐Parry 1986). Although different genera may dominate under local environmental conditions, our findings suggest that WWTP microbiomes across regions may functionally converge by supporting organisms that fulfill comparable ecological roles.

In contrast to bacteria, protists, and metazoa, influent fungal communities showed no clear north–south division in our dataset. Previous activated‐sludge studies suggest that fungal communities may respond less strongly to spatial distance than bacterial communities and may instead be structured by a combination of local wastewater characteristics, operational parameters, treatment process, temperature, nutrient load, and dispersal of abundant taxa (Wei et al. 2018; Zhang et al. 2018).

Together, our results demonstrate that both season and geographic location (latitude) shape microbial communities in WWTP influents, likely through active filtering processes that favor certain taxa while suppressing others, as well as through passive influences that promote seasonal, community‐wide dynamics. We were able to uncover biogeographic patterns in the dominance of individual taxa, revealing both site‐specific microbial changes and broader trends in community assembly. Importantly, our data confirm that bacterial and eukaryotic communities are not independent but mutually dependent, with evidence suggesting that shaping processes act bidirectionally across domains. Our findings emphasize that each treatment plant represents a distinct microbial ecosystem shaped by local environmental conditions. Because our dataset consists exclusively of sewage influent, we cannot directly infer the composition, assembly processes, or functional performance of resident activated‐sludge, biofilm, or effluent communities; nonetheless, our results underscore the need for regionally tailored monitoring and treatment strategies, as approaches effective in northern systems and monitoring protocols may not be directly transferable to southern WWTPs. The widespread occurrence of pathogenic genera in influent samples across regions also highlights the potential and relevance of wastewater‐based surveillance for public health monitoring. Furthermore, in light of ongoing climate change and increasingly pronounced seasonal extremes, close monitoring of WWTP microbiomes and timely responses to emerging challenges may become increasingly essential to ensure stable and effective treatment performance. Future research should further investigate bacterial–microeukaryotic interactions in WWTPs from non‐European and Southern Hemisphere regions to uncover broader biogeographic patterns and support the development of context‐specific solutions. Network analyses based on published data, such as this dataset, could be a useful way to investigate how microorganisms modulate community structure. Ultimately, viruses need to be included in further studies, although to comprehensively assess them DNA and RNA need to be collected (Weiss et al. 2026). Studies should also compare different treatment stages, ranging from influent through activated sludge to effluent, to track changes in community structure and interaction dynamics across the treatment process.

## Author Contributions

Niklas Nett: data curation, formal analysis, investigation, methodology, writing – original draft, writing – review and editing. Kenneth Dumack: conceptualization, formal analysis, project administration, supervision, writing – review and editing.

## Funding

This work was funded by the Deutsche Forschungsgemeinschaft (DFG, German Research Foundation), Project number 556896378, “Unlocking the Potential of Bioindicators in Wastewater Treatment,” awarded to K.D.

## Supporting information


**Figure S1:** Rarefaction curves for all WWTPs samples. Number of detected genera (y‐axis) mapped against the number of reads (x‐axis) in 100‐read steps, with genera assigned to five or fewer reads excluded.
**Figure S2:** Rarefaction curves for only Prokaryotes for all WWTPs samples. Number of detected genera (y‐axis) mapped against the number of reads (x‐axis) in 100‐read steps, with genera assigned to five or fewer reads excluded.
**Figure S3:** Rarefaction curves for only Eukaryotes for all WWTPs samples. Number of detected genera (y‐axis) mapped against the number of reads (x‐axis) in 100‐read steps, with genera assigned to five or fewer reads excluded.

**Table S1:** Sample metadata for untreated sewage influent samples. Metadata for all sewage influent samples reanalyzed in this study. The table includes collection date, WWTP‐associated sampling site, city, country, latitude, longitude, sampling type, sewage type, ENA sample accession, ENA alias, ENA run accession, and replicate identifier.

**Table S2:** Read counts and genus‐level richness of major microbial groups across influent sampling sites. Summary of taxonomic assignments for Bacteria, Archaea, Protists, Fungi, and microscopic Metazoa across the seven WWTP‐associated influent sampling sites.

**Table S3:** Alpha diversity and relative abundance of dominant genera in influent microbial communities. Sample‐level Shannon diversity indices and relative abundances of the 10 most abundant genera for each microbial group analyzed separately for Bacteria, Protists, Fungi, and microscopic Metazoa. Remaining genera are pooled as “Others.” Values are provided for each sample accession, sampling site, and sampling date.

**Table S4:** Global PERMANOVA results for factors associated with influent microbial community composition. PERMANOVA results based on Bray–Curtis dissimilarities of genus‐level community composition for Bacteria, Protists, Fungi, and microscopic Metazoa across all influent samples. Models include latitude, sampling site, season, and the first principal coordinate axis of the other microbial groups as explanatory variables.

**Table S5:** Site‐specific PERMANOVA results for seasonal and cross‐community associations in influent samples. PERMANOVA results calculated separately for each WWTP‐associated influent sampling site and each microbial group.

## Data Availability

Raw sequencing data were previously published by Becsei et al. (2024) and are available in the European Nucleotide Archive under accession PRJEB68319. The scripts, processed data, and Supporting Information tables used in this study are available at https://github.com/NiklasNett/whole‐wwtp‐microbiome‐europe.
