# Supplementary material for: A Pan‐European Whole‐Microbiome Study of Wastewater Influent: Prokaryotes, Protists, Fungi, and Metazoa
Source: J Eukaryot Microbiol. 2026 Sep 4;73(5):e70112. doi: 10.1111/jeu.70112 (PMC13543059; doi:10.1111/jeu.70112)
Supplement: Supplementary file 1 [file JEU-73-e70112-s006.docx]

Supplementary information

Whole Microbiome Analyses of Wastewater Treatment Plants

across Europe

Authors Niklas Nett^a^ and Kenneth Dumack^a^

Corresponding author Kenneth Dumack

Phone: +49-(0)221-470-6635 Fax: +49-(0)221-470-5038

^a^ University of Cologne, Terrestrial Ecology, Institute of Zoology, Zülpicher Str. 47b, 50674 Köln, Germany

nnett2@smail.uni-koeln.de, kenneth.dumack@uni-koeln.de

**Supplementary Figure 1: Rarefaction curves for all WWTPs samples.** Number of detected genera (y-axis) mapped against the number of reads (x-axis) in 100-read steps, with genera assigned to five or fewer reads excluded.

**Supplementary Figure 2: Rarefaction curves for only Prokaryotes for all WWTPs samples.** Number of detected genera (y-axis) mapped against the number of reads (x-axis) in 100-read steps, with genera assigned to five or fewer reads excluded.

**Supplementary Figure 3: Rarefaction curves for only Eukaryotes for all WWTPs samples.** Number of detected genera (y-axis) mapped against the number of reads (x-axis) in 100-read steps, with genera assigned to five or fewer reads excluded.

**Supplementary Table S1. Sample metadata for untreated sewage influent samples.**
Metadata for all sewage influent samples reanalyzed in this study. The table includes collection date, WWTP-associated sampling site, city, country, latitude, longitude, sampling type, sewage type, ENA sample accession, ENA alias, ENA run accession, and replicate identifier.

**Supplementary Table S2. Read counts and genus-level richness of major microbial groups across influent sampling sites.** Summary of taxonomic assignments for Bacteria, Archaea, Protists, Fungi, and microscopic Metazoa across the seven WWTP-associated influent sampling sites.

**Supplementary Table S3. Alpha diversity and relative abundance of dominant genera in influent microbial communities.** Sample-level Shannon diversity indices and relative abundances of the ten most abundant genera for each microbial group analyzed separately for Bacteria, Protists, Fungi, and microscopic Metazoa. Remaining genera are pooled as “Others.” Values are provided for each sample accession, sampling site, and sampling date.

**Supplementary Table S4. Global PERMANOVA results for factors associated with influent microbial community composition.** PERMANOVA results based on Bray–Curtis dissimilarities of genus-level community composition for Bacteria, Protists, Fungi, and microscopic Metazoa across all influent samples. Models include latitude, sampling site, season, and the first principal coordinate axis of the other microbial groups as explanatory variables.

**Supplementary Table S5. Site-specific PERMANOVA results for seasonal and cross-community associations in influent samples.** PERMANOVA results calculated separately for each WWTP-associated influent sampling site and each microbial group.
